# 4,4′-Bipyridine–terephthalic acid (1/1)

**DOI:** 10.1107/S1600536812021113

**Published:** 2012-05-12

**Authors:** Vandavasi Koteswara Rao, Matthias Zeller, Sherri R. Lovelace-Cameron

**Affiliations:** a Department of Chemistry, Youngstown State University, One University Plaza, Youngstown, OH 44555, USA

## Abstract

The asymmetric unit of the title compound, C_10_H_8_N_2_·C_8_H_6_O_4_, consists of one half-mol­ecule of each moiety, 4,4′-bipyridine (bpy) and terephthalic acid (bdc), both being located on crystallographic inversion centers. They are linked together *via* strong inter­molecular O—H⋯N hydrogen bonds, forming infinite chains propagating along [1-21]. The chains are further connected through C—H⋯O inter­actions giving sheets in (012). The sheets are linked *via* π–π inter­actions between the bpy rings and the bdc–bpy rings [centroid–centroid distances = 3.690 (2) and 3.869 (2) Å], resulting in the formation of a three-dimensional supra­molecular layer-like structure.

## Related literature

For dissolution of metal salts, see: Karpova *et al.* (2004 ▶); Yao *et al.* (2008 ▶); Zhao *et al.* (2007 ▶).
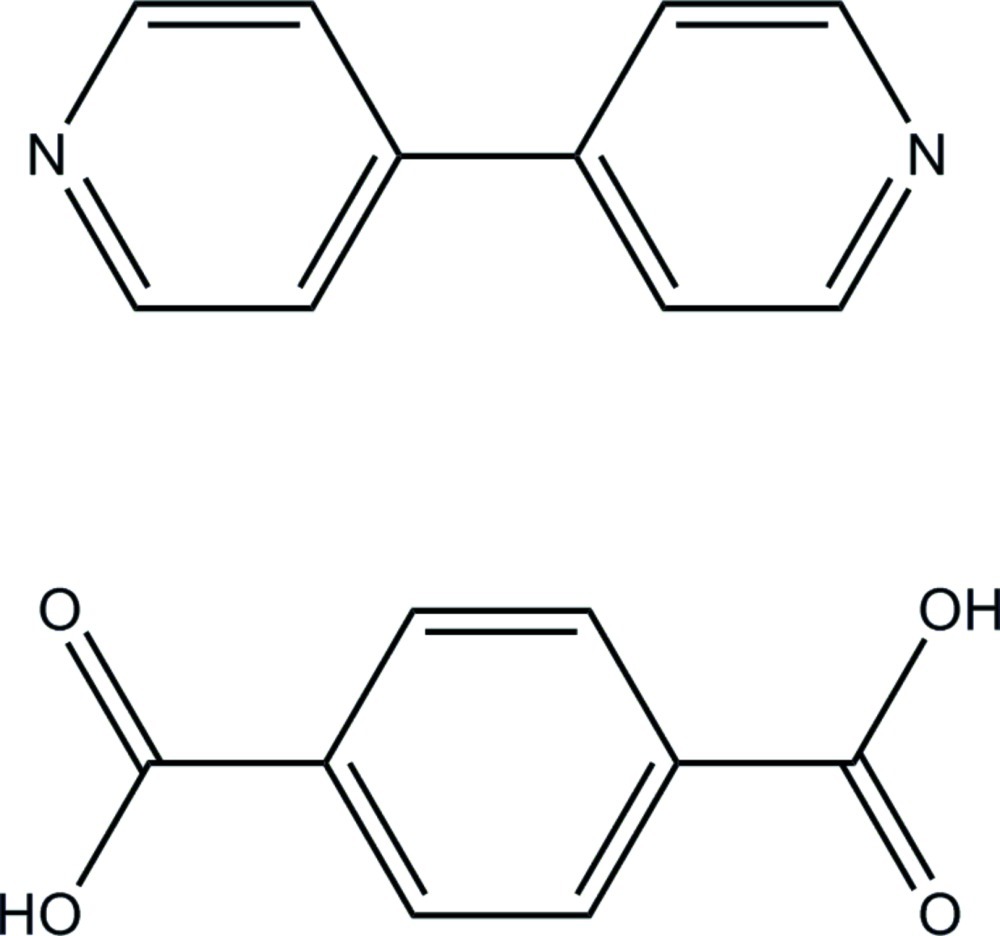


## Experimental

### 

#### Crystal data

C_10_H_8_N_2_·C_8_H_6_O_4_
*M* *_r_* = 322.31
Triclinic, 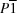

*a* = 6.783 (4) Å
*b* = 6.895 (4) Å
*c* = 8.161 (5) Å
α = 98.340 (8)°
β = 95.845 (9)°
γ = 104.623 (8)°
*V* = 361.5 (4) Å^3^
*Z* = 1
Mo *K*α radiation
μ = 0.11 mm^−1^
*T* = 100 K
0.39 × 0.21 × 0.13 mm

#### Data collection

Bruker SMART APEXII CCD diffractometer
Absorption correction: multi-scan (*SADABS*; Bruker, 2011 ▶) *T*_min_ = 0.659, *T*_max_ = 0.746
4318 measured reflections
2215 independent reflections
1750 reflections with *I* > 2σ(*I*)
*R*_int_ = 0.020

#### Refinement

*R*[*F* ^2^ > 2σ(*F* ^2^)] = 0.049
*wR*(*F* ^2^) = 0.143
*S* = 1.05
2215 reflections
111 parameters
H atoms treated by a mixture of independent and constrained refinement
Δρ_max_ = 0.43 e Å^−3^
Δρ_min_ = −0.33 e Å^−3^

### 

Data collection: *APEX2* (Bruker, 2011 ▶); cell refinement: *SAINT* (Bruker, 2011 ▶); data reduction: *SAINT*; program(s) used to solve structure: *SHELXS97* (Sheldrick, 2008 ▶); program(s) used to refine structure: *SHELXLE* (Hübschle *et al.*, 2011 ▶) and *SHELXL97* (Sheldrick, 2008 ▶); molecular graphics: *DIAMOND* (Brandenburg, 2001 ▶); software used to prepare material for publication: *publCIF* (Westrip, 2010 ▶).

## Supplementary Material

Crystal structure: contains datablock(s) I, global. DOI: 10.1107/S1600536812021113/su2418sup1.cif

Structure factors: contains datablock(s) I. DOI: 10.1107/S1600536812021113/su2418Isup2.hkl

Supplementary material file. DOI: 10.1107/S1600536812021113/su2418Isup3.cml

Additional supplementary materials:  crystallographic information; 3D view; checkCIF report

## Figures and Tables

**Table 1:** Hydrogen-bond geometry (Å, °)

*D*—H⋯*A*	*D*—H	H⋯*A*	*D*⋯*A*	*D*—H⋯*A*
O2—H2⋯N1^i^	1.10	1.49	2.5899 (17)	175
C5—H5⋯O1^ii^	0.95	2.50	3.231 (3)	134

Symmetry codes: (i) 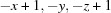; (ii) 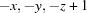.

**Fig. 1.**
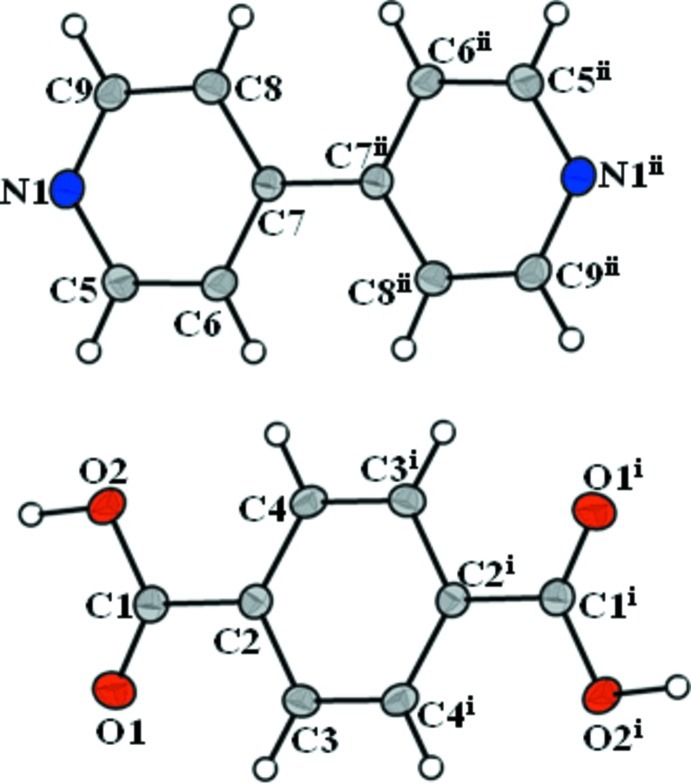
View of the title organic compound with atom numbering. Displacement ellipsoids are drawn at the 50% probability level [symmetry codes: (i) -x, -y + 1, -z + 1; (ii) -x + 1, -y + 1, -z].

**Fig. 2.**
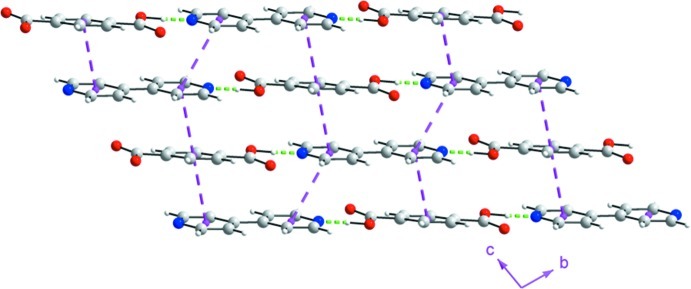
A view along the a axis of the two-dimensional supramolecular layer-like structure of the title compound. Green and purple dots represent the hydrogen bonds within the chains and the π···π stacking interactions between the chains, respectively [see Table 1 for details].

**Fig. 3.**
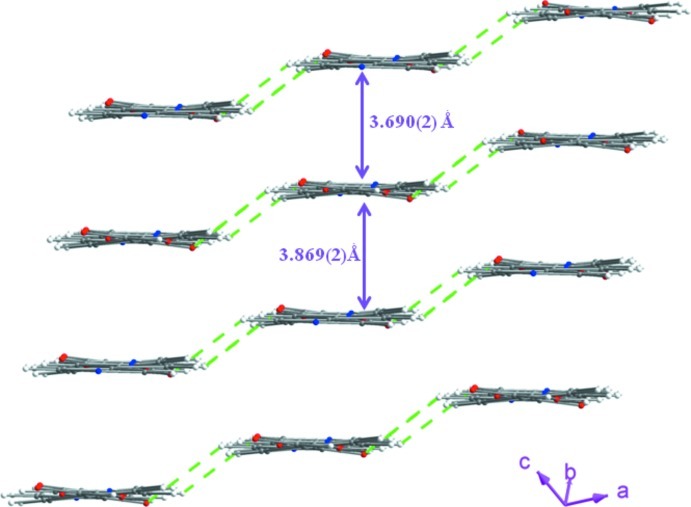
View of the three-dimensional supramolecular-like structure. Green dots represent the C-H···O hydrogen bonds connecting parallel stacks of chains. The purple arrows represent the π-π interactions within the π-stacks [see Table 1 for details].

## supplementary crystallographic information

### Comment

The title organic compound was obtained as a side-product during part of our investigations into the solvothermal synthesis of magnesium based metal organic framework compounds. Reaction of magnesium nitrate with terephthalic acid and 4,4'-bipyridine at 373 K did not yield an extended framework, but the high solubility of magnesium nitrate in DMF led to formation of the title organic compound in the form of colorless rod-like crystals. Similar dissolution of metal salts in organic solvents, leaving the organic compound as a side-product, have previously been observed (Karpova *et al.*, 2004; Yao *et al.*, 2008; Zhao *et al.*, 2007).

The asymmetric unit of the title organic compound is composed of one half-molecule of each component, that is 4,4'-bipyridine (bpy) and terephthalic acid (bdc). The inversion symmetry within bpy and bdc generates the full molecules (Fig. 1).

Strong inter-molecular O—H···N hydrogen bonds between the bpy and bdc molecules lead to the formation of infinite one-dimensional chains propagating along [1 -2 1] (Table 1 and Fig. 2). The pyridine rings of the bpy molecule exhibit a planar configuration with a dihedral angle of 0° between the planes of the two pyridyl rings. The dihedral angle between the bpy and bdc molecules within the chains is 7.20 (4)°.

The bpy molecules and the bdc molecules in neighbouring chains are π-stacked above one another [centroid-centroid distance of 3.869 (2) Å; symmetry code: -x+1, -y, -z] in an *ABAB*···fashion resulting in a two-dimensional supramolecular layer-like structure (Fig. 2). The interplanar spacing between the chains, defined as the distance between the planes of neighbouring bpy rings is 3.2512 Å [centroid-centroid distance 3.690 (2) Å; slippage of 1.745 Å]. The layers, formed through π-stacking, are further connected through C-H···O interactions along (012) resulting in a three-dimensional supramolecular-like structure (Fig. 3). Thus the overall structure of the title compound is stabilized by strong hydrogen bonds and π-π interactions.

### Experimental

The compound was synthesized under solvothermal conditions. In a typical synthesis, Mg(NO_3_)_2_.6H_2_O (0.129 g, 1.0 mmol) and terepthalic acid (0.169 g, 2.0 mmol) were dissolved in DMF (5.0 ml). Then, 4,4'-bipyridine (0.239 g, 3.0 mmol) was added to the reaction mixture and stirred for one hour before transferring the mixture into a glass vial. The final mixture was heated to 373 K (100 °C) for 24 h. The vial was then slowly cooled to room temperature. Slow cooling of the reaction mixture yielded colorless rod-like crystals of the title compound as a minor product, along with an unidentified white powder.

### Refinement

The carboxylic acid H atoms were located in difference electron density maps, but were placed in calculated positions with fixed C—O—H angles, but with the C—C—O—H dihedral angles and the O—H distances freely refined (AFIX 148 command in *SHELXTL*; Sheldrick, 2008). The C-bound H atoms were included in calculated positions: C-H = 0.95 Å; *U*_iso_(H) = k × *U*_eq_(O,C), where k = 1.5 for carboxylic acid H atoms, and = 1.2 for other H atoms.

### Crystal data

**Table:**

C_10_H_8_N_2_·C_8_H_6_O_4_	*Z* = 1
*M**_r_* = 322.31	*F*(000) = 168
Triclinic, *P*1	*D*_x_ = 1.480 Mg m^−^^3^
Hall symbol: -P 1	Mo *K*α radiation, λ = 0.71073 Å
*a* = 6.783 (4) Å	Cell parameters from 245 reflections
*b* = 6.895 (4) Å	θ = 4.3–31.4°
*c* = 8.161 (5) Å	µ = 0.11 mm^−^^1^
α = 98.340 (8)°	*T* = 100 K
β = 95.845 (9)°	Rod, colorless
γ = 104.623 (8)°	0.39 × 0.21 × 0.13 mm
*V* = 361.5 (4) Å^3^	

### Data collection

**Table:**

Bruker SMART APEXII CCD diffractometer	2215 independent reflections
Radiation source: fine-focus sealed tube	1750 reflections with *I* > 2σ(*I*)
Graphite monochromator	*R*_int_ = 0.020
ω scans	θ_max_ = 31.5°, θ_min_ = 2.6°
Absorption correction: multi-scan (*SADABS*; Bruker, 2011)	*h* = −9→9
*T*_min_ = 0.659, *T*_max_ = 0.746	*k* = −10→9
4318 measured reflections	*l* = −11→11

### Refinement

**Table:**

Refinement on *F*^2^	Primary atom site location: structure-invariant direct methods
Least-squares matrix: full	Secondary atom site location: difference Fourier map
*R*[*F*^2^ > 2σ(*F*^2^)] = 0.049	Hydrogen site location: inferred from neighbouring sites
*wR*(*F*^2^) = 0.143	H atoms treated by a mixture of independent and constrained refinement
*S* = 1.05	*w* = 1/[σ^2^(*F*_o_^2^) + (0.0819*P*)^2^ + 0.0211*P*] where *P* = (*F*_o_^2^ + 2*F*_c_^2^)/3
2215 reflections	(Δ/σ)_max_ < 0.001
111 parameters	Δρ_max_ = 0.43 e Å^−^^3^
0 restraints	Δρ_min_ = −0.33 e Å^−^^3^

### Special details

**Table:**

Geometry. All e.s.d.'s (except the e.s.d. in the dihedral angle between two l.s. planes) are estimated using the full covariance matrix. The cell e.s.d.'s are taken into account individually in the estimation of e.s.d.'s in distances, angles and torsion angles; correlations between e.s.d.'s in cell parameters are only used when they are defined by crystal symmetry. An approximate (isotropic) treatment of cell e.s.d.'s is used for estimating e.s.d.'s involving l.s. planes.
Refinement. Refinement of *F*^2^ against ALL reflections. The weighted *R*-factor *wR* and goodness of fit *S* are based on *F*^2^, conventional *R*-factors *R* are based on *F*, with *F* set to zero for negative *F*^2^. The threshold expression of *F*^2^ > σ(*F*^2^) is used only for calculating *R*-factors(gt) *etc*. and is not relevant to the choice of reflections for refinement. *R*-factors based on *F*^2^ are statistically about twice as large as those based on *F*, and *R*- factors based on ALL data will be even larger.

### Fractional atomic coordinates and isotropic or equivalent isotropic displacement parameters (Å^2^)

**Table:**

	*x*	*y*	*z*	*U*_iso_*/*U*_eq_	
C1	0.11190 (17)	0.19752 (17)	0.67373 (14)	0.0175 (2)	
C2	0.05394 (16)	0.35241 (16)	0.58115 (13)	0.0155 (2)	
C3	−0.14077 (17)	0.38403 (17)	0.58505 (14)	0.0178 (2)	
H3	−0.2370	0.3048	0.6427	0.021*	
C4	0.19359 (17)	0.46838 (17)	0.49560 (14)	0.0173 (2)	
H4	0.3260	0.4464	0.4924	0.021*	
C5	0.44781 (18)	0.16270 (17)	0.21492 (14)	0.0198 (2)	
H5	0.3570	0.0943	0.2824	0.024*	
C6	0.39356 (17)	0.31190 (17)	0.13785 (14)	0.0187 (2)	
H6	0.2664	0.3421	0.1512	0.022*	
C7	0.52575 (16)	0.41778 (16)	0.04063 (13)	0.0152 (2)	
C8	0.70734 (17)	0.36148 (17)	0.02350 (14)	0.0181 (2)	
H8	0.8020	0.4280	−0.0422	0.022*	
C9	0.74945 (17)	0.20858 (17)	0.10241 (14)	0.0186 (2)	
H9	0.8731	0.1714	0.0881	0.022*	
N1	0.62329 (15)	0.11110 (14)	0.19795 (12)	0.0181 (2)	
O1	0.00764 (13)	0.12024 (14)	0.77275 (11)	0.0266 (2)	
O2	0.28465 (13)	0.15786 (13)	0.63990 (11)	0.0228 (2)	
H2	0.3174 (12)	0.044 (2)	0.7131 (16)	0.034*	

### Atomic displacement parameters (Å^2^)

**Table:**

	*U*^11^	*U*^22^	*U*^33^	*U*^12^	*U*^13^	*U*^23^
C1	0.0174 (5)	0.0182 (5)	0.0173 (5)	0.0061 (4)	0.0004 (4)	0.0040 (4)
C2	0.0166 (5)	0.0167 (5)	0.0140 (5)	0.0060 (4)	0.0013 (4)	0.0032 (4)
C3	0.0165 (5)	0.0207 (5)	0.0181 (5)	0.0056 (4)	0.0042 (4)	0.0067 (4)
C4	0.0146 (5)	0.0214 (5)	0.0182 (5)	0.0079 (4)	0.0030 (4)	0.0047 (4)
C5	0.0202 (5)	0.0197 (5)	0.0214 (5)	0.0060 (4)	0.0046 (4)	0.0074 (4)
C6	0.0169 (5)	0.0207 (5)	0.0216 (5)	0.0086 (4)	0.0043 (4)	0.0065 (4)
C7	0.0152 (5)	0.0153 (5)	0.0149 (5)	0.0048 (4)	0.0002 (4)	0.0025 (4)
C8	0.0162 (5)	0.0205 (5)	0.0195 (5)	0.0063 (4)	0.0030 (4)	0.0064 (4)
C9	0.0159 (5)	0.0200 (5)	0.0214 (5)	0.0080 (4)	0.0010 (4)	0.0042 (4)
N1	0.0187 (5)	0.0179 (5)	0.0184 (5)	0.0069 (4)	−0.0001 (4)	0.0037 (3)
O1	0.0230 (4)	0.0325 (5)	0.0316 (5)	0.0112 (4)	0.0088 (4)	0.0193 (4)
O2	0.0222 (4)	0.0274 (5)	0.0267 (5)	0.0150 (3)	0.0080 (3)	0.0126 (3)

### Geometric parameters (Å, º)

**Table:**

C1—O1	1.2198 (14)	C5—H5	0.9500
C1—O2	1.3152 (15)	C6—C7	1.3960 (16)
C1—C2	1.5004 (17)	C6—H6	0.9500
C2—C4	1.3922 (16)	C7—C8	1.3957 (16)
C2—C3	1.3945 (17)	C7—C7^ii^	1.489 (2)
C3—C4^i^	1.3883 (16)	C8—C9	1.3853 (16)
C3—H3	0.9500	C8—H8	0.9500
C4—C3^i^	1.3883 (16)	C9—N1	1.3377 (15)
C4—H4	0.9500	C9—H9	0.9500
C5—N1	1.3399 (16)	O2—H2	1.1019
C5—C6	1.3859 (17)		
			
O1—C1—O2	124.24 (11)	C5—C6—C7	120.01 (11)
O1—C1—C2	121.98 (11)	C5—C6—H6	120.0
O2—C1—C2	113.77 (10)	C7—C6—H6	120.0
C4—C2—C3	119.74 (10)	C8—C7—C6	116.69 (10)
C4—C2—C1	120.93 (10)	C8—C7—C7^ii^	121.97 (12)
C3—C2—C1	119.31 (10)	C6—C7—C7^ii^	121.34 (12)
C4^i^—C3—C2	119.79 (10)	C9—C8—C7	119.94 (10)
C4^i^—C3—H3	120.1	C9—C8—H8	120.0
C2—C3—H3	120.1	C7—C8—H8	120.0
C3^i^—C4—C2	120.48 (11)	N1—C9—C8	122.74 (11)
C3^i^—C4—H4	119.8	N1—C9—H9	118.6
C2—C4—H4	119.8	C8—C9—H9	118.6
N1—C5—C6	122.57 (11)	C9—N1—C5	118.02 (10)
N1—C5—H5	118.7	C1—O2—H2	109.5
C6—C5—H5	118.7		
			
O1—C1—C2—C4	166.89 (11)	N1—C5—C6—C7	−1.27 (18)
O2—C1—C2—C4	−11.94 (15)	C5—C6—C7—C8	1.53 (17)
O1—C1—C2—C3	−11.33 (17)	C5—C6—C7—C7^ii^	−178.58 (12)
O2—C1—C2—C3	169.83 (10)	C6—C7—C8—C9	−0.57 (17)
C4—C2—C3—C4^i^	−0.35 (18)	C7^ii^—C7—C8—C9	179.53 (11)
C1—C2—C3—C4^i^	177.90 (10)	C7—C8—C9—N1	−0.75 (18)
C3—C2—C4—C3^i^	0.35 (18)	C8—C9—N1—C5	1.07 (17)
C1—C2—C4—C3^i^	−177.87 (10)	C6—C5—N1—C9	−0.05 (17)

Symmetry codes: (i) −*x*, −*y*+1, −*z*+1; (ii) −*x*+1, −*y*+1, −*z*.

### Hydrogen-bond geometry (Å, º)

**Table:**

*D*—H···*A*	*D*—H	H···*A*	*D*···*A*	*D*—H···*A*
O2—H2···N1^iii^	1.10	1.49	2.5899 (17)	175
C5—H5···O1^iv^	0.95	2.50	3.231 (3)	134

Symmetry codes: (iii) −*x*+1, −*y*, −*z*+1; (iv) −*x*, −*y*, −*z*+1.
